# Supplementary material for: WordCommentsAnalyzer: A windows software tool for qualitative research
Source: F1000Res. 2018 Sep 4;7:536. Originally published 2018 May 3. [Version 2] doi: 10.12688/f1000research.14819.2 (PMC6137411; doi:10.12688/f1000research.14819.2)
Supplement: Supplementary file 3 [file f1000research-7-17614-s0002.tgz › b2a79496-4ada-47c4-9682-a99ce867836d.zip!/Eight Iranian Health Journals/Yafteh _0.docx]

© Yafteh  . This work is licensed under CC BY 4.0 (https://creativecommons.org/licenses/by/4.0/).

Volume & Issue: دوره 20 - شماره 1

Title: رابطه بین وابستگی به تلفن همراه با ویژگی‌های تحصیلی دانشجویان دانشگاه علوم پزشکی لرستان

http://yafte.lums.ac.ir/article-1-2424-fa.html

مقدمه: تلفن همراه علاوه بر داشتن کاربردهای گسترده می‌تواند موجب بروز برخی پیامدهای نامناسب شود. با توجه به رواج روزافزون استفاده از این فناوری در دانشجویان، پژوهش حاضر با هدف تعیین سطح وابستگی به تلفن همراه و ارتباط آن با ویژگی­ های تحصیلی دانشجویان دانشگاه علوم پزشکی لرستان انجام شد. مواد و روش ­ها: این مطالعه توصیفی تحلیلی در جامعه دانشجویان دانشکده­ های پیراپزشکی، بهداشت و تغذیه دانشگاه علوم پزشکی لرستان در نیمسال اول تحصیلی 95-94 انجام شد. روش نمونه­ گیری تصادفی طبقه ­ای بود. داده ­ها با استفاده از پرسشنامه وابستگی به تلفن همراه (CPDQ) جمع ­آوری شد که ویژگی­ های روانسنجی آن مورد تأیید قرار گرفته بود. تحلیل داده­ ها با نرم‌افزار SPSS و آزمون­ های آماری t مستقل، ضریب همبستگی پیرسون، آنالیز واریانس و آزمون تعقیبی دانکن انجام شد. یافته ­ها: میانگین نمره وابستگی به تلفن همراه در دانشجویان 08/8 ± 61/40 و در سطح متوسط بود. یافته ­های حاصل از پژوهش نشان داد که 7/24 درصد از شرکت‌ کنندگان در مطالعه وابستگی خفیف، 3/51 درصد وابستگی متوسط و 24 درصد وابستگی شدید به تلفن همراه داشتند. همچنین، بین نمره وابستگی به تلفن همراه و معدل نیمسال قبل (03/0=, P19/0-=r)   و معدل کل (006/0=, P2/0-=r) رابطه معکوس معناداری وجود داشت. بحث و نتیجه­ گیری: یافته ­های مطالعه نشان داد که وابستگی به تلفن همراه موجب کاهش موفقیت تحصیلی می‌شود. با توجه به گسترش روزافزون استفاده از تلفن همراه در بین دانشجویان، انجام برنامه ­ریزی و کنترل­ های لازم برای جلوگیری از پیامدهای منفی آن ضروری است.

Title: بررسی مقایسه اثرات ضدقارچی عصاره‌‌های مختلف گیاه Onosma Chlorotricum بر قارچ‌های کاندیدا آلبیکنس و کاندیدا گلابراتا با دو آنتی‌بیوتیک فلوکونازول و نیستاتین

http://yafte.lums.ac.ir/article-1-2521-fa.html

مقدمه: با توجه به عود عفونت‌های کاندیدائی و مقاومت آن‌ها به داروهای ضدقارچی در طی درمان و نیز عوارض شناخته شده داروهای شیمیایی و وجود بعضی تحقیقات مبنی بر اینکه جنس Onosma حاوی مشتقات آلکانین و شیکونین بوده که دارای خواص ضدقارچی می‌باشند، برای اولین بار خواص ضد قارچی گیاه Onosma Chlorotricum در مقایسه با آنتی‌بیوتیک‌ مورد بررسی قرار گرفت. مواد و روش ­ها: گیاه مذکور در اواسط تیرماه از شهرستان کوهدشت جمع‌ آوری و پس از شناسایی و خشک کردن، عصاره‌های ان-هگزانی، متانولی و آبی به روش خیساندن تهیه شد. اثرات ضدقارچی غلظت‌های مختلف عصاره‌ها با استفاده از روش انتشار دیسک و MIC (حداقل غلظت ممانعت از رشد)، MFC (حداقل غلظت قارچ‌کشی) به روش میکرودایلوشن بررسی شد. آنتی‌بیوتیک فلوکونازول و نیستاتین به عنوان کنترل مثبت، DMSO به عنوان کنترل منفی استفاده گردید. داده‌ها توسط آزمون t و واریانس یک‌ طرفه ANOVA آنالیز شدند. یافته­ ها: میانگین قطر هاله عدم رشد عصاره ان-هگزانی در غلظت 10 میلی‌گرم بر میلی‌لیتر بیشتر از قطر هاله عدم رشد آنتی‌بیوتیک نیستاتین برای کاندیدا آلبیکنس و گلابراتا می‌باشد (05/0˂P). کمترین میزان MIC مربوط به عصاره ان-هگزانی برای کاندیدا آلبیکنس و گلابراتا به ترتیب برابر با μg/μl 62/15 و μg/μl 25/31، کمترین میزان MFC مربوط به عصاره ان-هگزانی برای کاندیدا آلبیکنس و گلابراتا به ترتیب برابر با μg/μl  25/31 و  μg/μl5/62 می‌باشد. بحث و نتیجه­ گیری: عصاره‌های این گیاه اثر ضدقارچی مناسبی بر کاندیدا آلبیکنس و گلابراتا نشان داد. بررسی احتمال کاربرد این عصاره در درمان عفونت کاندیدایی نیاز به اتمام مطالعات تکمیلی دارد.

Title: بررسی اختلالات اسکلتی عضلانی به روش QEC در بین جوشکاران شهر خرم‌آباد در سال 1394

http://yafte.lums.ac.ir/article-1-2463-fa.html

مقدمه: بر اساس تحقیقاتی که اخیراً انجام گرفته است، علت بیش از نیمی از غیبت‌های محیط کار و دلیل یک‌سوم درخواست غرامت‌های ناشی از کار را اختلالات اسکلتی عضلانی تشکیل می‌دهند. این مطالعه با هدف بررسی اختلالات اسکلتی عضلانی و ارزیابی ریسک ابتلا به این اختلالات در جوشکاران شهر خرم‌آباد انجام شده است. مواد و روش ­ها: این مطالعه توصیفی تحلیلی به روش سرشماری بر روی 170 نفر از جوشکاران شهر خرم‌آباد انجام شده است. میزان شیوع علائم اختلالات اسکلتی عضلانی با استفاده از پرسشنامه نوردیک بررسی شد. به منظور ارزیابی خطر ابتلا به اختلالات اسکلتی عضلانی از روش QEC استفاده شد. آنالیز داده‌ها با استفاده از شاخص آمار مرکزی و آزمون t انجام گرفت. یافته­ ها: نتایج مطالعه نشان داد که شیوع اختلالات اسکلتی عضلانی به ترتیب در نواحی کمر (70%)، زانو (1/54%) و تنه (50%) بالاترین میزان را دارد. نتایج ارزیابی به روش QEC نشان داد که 8/83% از موارد، سطح ریسک ابتلا به اختلالات بالا و بسیار بالا دارند. بحث و نتیجه ­گیری: این مطالعه نشان داد که شیوع اختلالات اسکلتی عضلانی در بین جوشکاران مورد مطالعه بالا است و همچنین ریسک ابتلا به این اختلالات نیز بالا می‌باشد. بر اساس نتایج به دست آمده، از علل ابتلا به اختلالات اسکلتی عضلانی در شغل جوشکاری می‌توان به پوسچر نامطلوب، بلند کردن و حمل دستی بار و خمش و پیچش کمر اشاره کرد. افزون بر این نتایج، این مطالعه نشان داد که روش QEC می‌تواند نتایج قابل اعتمادی را ارائه دهد.

Title: بررسی فنوتیپ و ژنوتیپ دو ژنVIM-I و IMP-Iمتالوبتالاکتاماز و الگوی مقاومت آنتی‌بیوتیکی در سودوموناس آئروژینوزا جدا شده از نمونه‌های بالینی بیمارستان شهید بهشتی شهرستان قم

http://yafte.lums.ac.ir/article-1-2456-fa.html

مقدمه: سودوموناس آئروژینوزا یک باسیل گرم منفی، اکسیداز مثبت، کاتالاز مثبت، غیرتخمیری، متحرک، هوازی اجباری و فاقد توانایی تخمیر کربوهیدرات است.  این باکتری یک پاتوژن فرصت طلب در بیماران دچار اختلال سیستم دفاعی و یکی از مرسوم ترین عوامل عفونت های بیمارستانی به شمار می‌آید.  از آنجا که در برابر طیف وسیعی از آنتی‌بیوتیک‌ها ازجمله پنی سیلین‌ها، سفالوسپورین‌های وسیع الطیف و کارباپنم‌ها (به استثنای مونوباکتام) مقاوم‌اند، یکی از مشکلات عمده بالینی به شمار می‌روند. VIM و  IMP  ازجمله ژن‌های پلاسمیدی متالوبتالاکتاماز هستند. هدف از این مطالعه بررسی فنوتیپ و ژنوتیپ ژن‌های متالوبتالاکتاماز VIM-I و IMP-I در جدایه‌های بالینی سودوموناس آئروژینوزا مقاوم به آنتی بیوتیک ایمی‌پنم از خانواده کارباپنم می‌باشد. مواد و روش ­ها: در این مطالعه، به واسطه کشت در محیط‌های عمومی و افتراقی و انجام تست‌های استاندارد بیوشیمیایی جداسازی صورت گرفت و برای تایید قطعی و بررسی ژنهای متالوبتالاکتاماز از PCR  بهره گرفته شد. برای بررسی مقاومت آنتی بیوتیکی نمونه‌ها و بررسی فنوتیپی ژنهای متالوبتالاکتاماز از دیسک دیفیوژن استفاده شد. یافته ­ها: گروه مورد مطالعه، ایزوله‌های سودوموناس آئروژینوزا گردآوری شده از بیمارستان شهید بهشتی شهرستان قم بود. از 100 سویه سودوموناس آئروژینوزا مورد مطالعه که جنس و گونه آن­ها بیشتر به واسطه تست­ های بیوشیمیایی تأیید شده بود، 48 نمونه مولد متالوبتالاکتاماز بود که از این تعداد، 19 نمونه (58/39%) دارای باندهایی با 587 جفت‌باز مربوط به ژن IMP، 6 نمونه (5/12%) دارای باندهایی با 261 جفت‌باز مربوط به ژن VIM و 3 نمونه (25/6%) حاوی هر دو ژن بودند. همچنین 36 نمونه (74%) نسبت به جنتامایسین، 30 نمونه (62%) نسبت به سیپروفلوکساسین ، 29 نمونه (61%) نسبت به سفتازیدیم، 27  نمونه (58%) نسبت به سفتی­زوکسیم و 19 نمونه (39%) نسبت به ایمی­پنم مقاوم بودند. بحث و نتیجه ­گیری: در این مطالعه 48 نمونه (48%)  از سویه ­های سودوموناس آئروژینوزا مولد MBL بودند. از این 48 جدایه، 6  نمونه (5/12%) دارای ژن VIM، 19 نمونه (58/39%)  دارای ژن IMP و 3 نمونه (25/6%) دارای هر دو ژن بودند. در مقایسه ژن IMP به تنهایی از آزمون فیشر رابطه معناداری یافت شد (05/0P≤). در حالی که در فنوتیپ و ژن  VIM به تنهایی از آزمون فیشر، رابطه معناداری وجود نداشت. درنتیجه نوع عفونت و منطقه و نوع آنتی‌بیوتیک‌های استفاده شده در بخش، می‌تواند در بیان ژن‌های متالوبتالاکتاماز تاثیرگذار باشد.

Title: بررسی استفاده از فرآیند الکتروفنتون با الکترودهای آهن و گرافیت برای حذف مترونیدازول از محلول های آبی

http://yafte.lums.ac.ir/article-1-2123-fa.html

مقدمه: استفاده بی‌رویه از آنتی‌بیوتیک‌ها و تخلیه آن­ها به محیط زیست پیامد­­­های جدی و خطرناکی را به دنبال دارد، از این‌رو حذف این آلاینده­ ها از محیط ­های آبی از ضرورت­ های مهم به شمار می‌آید. مترونیدازول نیز به‌عنوان یک آنتی‌ بیوتیک از جمله این آلاینده‌هاست که باید از محیط حذف گردد. از این‌رو هدف از این مطالعه بررسی اثربخشی روش اکسیداسیون پیشرفته الکتروفنتون در حذف مترونیدازول از محیط آبی بود. مواد و روش ­ها: این مطالعه تجربی و در مقیاس آزمایشگاهی بود که در آن تأثیر فاکتورهایی مانند pH محلول، زمان واکنش، غلظت اولیه مترونیدازول، جریان الکتریکی و غلظت پراکسیدهیدروژن بر روی راندمان حذف مترونیدازول بررسی شد. یافته­ ها: نتایج نشان داد که مقدار پراکسیدهیدروژن، شدت جریان و زمان واکنش دارای اثر مثبت و افزایش غلظت اولیه مترونیدازول دارای اثر منفی بر کارایی حذف مترونیدازول بودند. در مقادیر مناسب متغیر­ها 7 pH =، شدت جریان 30 ولت، زمان واکنش 40 دقیقه، دوز پراکسیدهیدروژن 02/0 مول در لیتر و غلظت اولیه مترونیدازول 10 میلی‌گرم در لیتر بیش از 99% مترونیدازول حذف گردید. بحث و نتیجه­ گیری: فرآیند الکتروفنتون در شرایط مناسب محیطی قادر به حذف مؤثر آنتی‌بیوتیک مترونیدازول از محیط ­های آبی بود و از این فرآیند می‌توان به‌عنوان یک روش مناسب برای حذف سایر آلاینده­ های مقاوم از محیط استفاده نمود.

Title: نقش محافظتی اتیل پیروات و ویتامین E در برابر سمیت کلیوی ناشی از فنیل‌هیدرازین در موش سفید کوچک آزمایشگاهی

http://yafte.lums.ac.ir/article-1-2491-fa.html

مقدمه: فنیل ­هیدرازین به ­عنوان یک ترکیب همولیتیک موجب سمیت در بافت­ های متعدد می­ گردد. مطالعه حاضر به­ منظور ارزیابی اثرات محافظتی احتمالی اتیل ­پیروات و ویتامین E در برابر آسیب­های کلیوی ناشی از فنیل­ هیدرازین در موش انجام پذیرفت. مواد و روش­ ها: موش ­های نر بالغ به صورت تصادفی به هشت گروه هشت سری تقسیم شدند. گروه کنترل سرم فیزیولوژی             (ml, IP 1/0) به طور روزانه دریافت نمودند. گروه دوم فنیل­ هیدرازین با دوز h48 mg/kg/60 به ­صورت داخل صفاقی دریافت نمودند. گروه سوم به همراه فنیل­ هیدرازین، ویتامین E با دوز mg/kg/day 100 به ­صورت داخل صفاقی دریافت نمودند. گروه چهارم به همراه فنیل‌هیدرازین، اتیل ­پیروات با دوز mg/kg/day 40 به ­صورت داخل صفاقی دریافت کردند. گروه پنجم به همراه فنیل­هیدرازین، ویتامین E و اتیل ­پیروات با دوز مشابه گروه ­های قبلی، دریافت کردند. گروه ششم ویتامین E، گروه هفتم اتیل­ پیروات و گروه هشتم اتیل ­پیروات و ویتامین E با دوزهای مشابه گروه­ های قبلی بدون تزریق فنیل­ هیدرازین دریافت نمودند. پس از 35 روز نمونه­ های سرمی و بافتی تهیه شده و جهت ارزیابی ­های بیوشیمیایی، بافت ­شناسی و هیستومورفومتری مورد استفاده قرار گرفتند. یافته­ ها: فنیل­هیدرازینبه شکل معنی ­داری موجب افزایش سطحسرمی مالون ­دی ­آلدئید، کراتینین، اوره و نیز کاهش ظرفیت آنتی‌اکسیدانت تام سرم و آلبومین گردید (05/0P<). به‌علاوه، فنیل­ هیدرازین افزایش معنی­ داری (05/0P<) در قطر حفره میانی لوله­های پیچیده نزدیک و نیز کاهش معنی ­داری (05/0P<) در ارتفاع سلول ­های پوششی این لوله ­ها ایجاد کرد. تجویز ویتامین E و اتیل­پیروات به شکل قابل توجهی تغییرات مشاهده شده در فراسنجه­ های مذکور را بهبود بخشید. بحث و نتیجه ­گیری: به نظر می ­رسد اتیل ­پیروات و ویتامین E به عنوان یک مهار­کننده رادیکال آزاد می­تواند سمیت کلیوی ناشی از فنیل­ هیدرازین در موش را کاهش دهد.

Title: تأثیر هشت هفته فعالیت استقامتی بر بیان ژن‌های‌ فاکتور افزایش‌دهنده مایوسیت-2 و هیستون داستیلاز4 عضلات اسکلتی کند و تند انقباض موش‌های صحرایی نر ویستار

http://yafte.lums.ac.ir/article-1-2510-fa.html

مقدمه: عضلات اسکلتی از تارهایی با انقباضات مختلف تشکیل‌ شده‌اند که به‌ طور عمده به تارهای کند انقباض و تند انقباض تقسیم می‌شوند. هدف از این پژوهش بررسی تأثیر هشت هفته فعالیت استقامتی بر بیان ژن فاکتور افزایش‌دهنده مایوسیت-2 و هیستون داستیلاز4 در عضلات اسکلتی نوع کند و تند انقباض موش‌های صحرایی نر ویستار است. مواد و روش ­ها: بدین منظور 20 سر موش صحرایی نر ویستار با 4 هفته سن از مرکز تحقیقات رازی دانشگاه علوم پزشکی لرستان خریداری شد. شرایط آزمایشگاهی یکسان برای موش‌ها تا رسیدن به سن بلوغ فراهم شد. آشناسازی استقامتی 14 روزه برای آشنایی با دویدن روی تردمیل صورت گرفت. در پایان دوره، موش‌ها به‌صورت تصادفی به دو گروه تجربی (ده سر) و گروه کنترل (ده سر) تقسیم شدند. یک برنامه استقامتی 8 هفته‌ای، 5 جلسه در هفته برای گروه تجربی اجرا شد. یافته­ ها: این پژوهش نشان داد بیان نسبی ژن هیستون داستیلاز4 در عضله بازکننده دراز انگشتان پا تمرینی در مقایسه با گروه شاهد به لحاظ آماری تغییر معناداری نداشت و بیان نسبی ژن فاکتور افزایش‌دهنده مایوسیت-2 در عضله بازکننده دراز انگشتان پا گروه تمرینی نسبت به گروه شاهد علی‌رغم کاهش، به لحاظ آماری معنی‌دار نبود. در عضله نعلی بیان نسبی ژن هیستون داستیلاز4 در گروه تمرینی نسبت به گروه شاهد از لحاظ آماری تغییر معناداری مشاهده نگردید. اما بیان نسبی ژن فاکتور افزایش‌دهنده مایوسیت-2 در گروه تمرینی در مقایسه با گروه شاهد به لحاظ آماری کاهش معنی‌داری داشت (05/0P<). بحث و نتیجه­ گیری: به‌طور خلاصه نتایج این پژوهش نشان داد که فعالیت استقامتی به مدت 8 هفته موجب تغییر در بیان ژن‌های هیستون داستیلاز4 و فاکتور افزایش‌دهنده مایوسیت-2 در عضله بازکننده دراز انگشتان پا نگردید اما در عضله نعلی، بیان ژن فاکتور افزایش‌ دهنده  مایوسیت-2 کاهش یافت همچنین در میزان بیان ژن هیستون داستیلاز4 نیز تغییری مشاهده نشد.

Title: بررسی فراوانی IgM و IgG توکسوپلاسما گوندی در بیماران همودیالیزی مراکز منتخب همودیالیز شهر تهران در سال 1395

http://yafte.lums.ac.ir/article-1-2507-fa.html

مقدمه: توکسوپلاسموزیس از جمله بیماری‌های مشترک بین انسان و حیوان است که در اثر آلودگی با انگل تک یاخته درون سلولی توکسوپلاسما گوندی ایجاد می‌شود. آلودگی به این انگل عمدتاً فاقد علامت بالینی است ولی در افراد با ضعف سیستم ایمنی مانند افراد تحت شیمی درمانی، بیماران پیوندی، سرطانی و ایدزی و هم‌چنین افراد دیالیزی می‌تواند عوارض شدیدی داشته باشد. با توجه به شیوع بالای انگل در نقاط مختلف ایران، بر آن شدیم تا به بررسی میزان شیوع توکسوپلاسموزیس در افراد تحت همودیالیز در مراکز منتخب شهر تهران بپردازیم. مواد و روش ­ها: در این مطالعه توصیفی - مقطعی، 260 بیمار همودیالیزی مراجعه کننده به 5 مرکز دیالیز شهر تهران در سال 95 مورد مطالعه قرار گرفتند. نمونه سرم‌های جمع‌آوری شده از نظر وجود آنتی‌بادی‌های IgG و IgM ضد توکسوپلاسما به روش الایزا مورد بررسی قرار گرفتند. یافته­ ها: در این مطالعه 175 نفر (3/67%) از بیماران همودیالیزی مورد مطالعه از نظر آنتی‌بادی IgG و 18 نفر (7%) از نظر آنتی‌بادی IgM ضد توکسوپلاسما مثبت بودند. بحث و نتیجه­ گیری: با توجه به شیوع بالای عفونت توکسوپلاسموزیس در بین بیماران همودیالیزی شهر تهران در مقایسه با سایر افراد این منطقه و با در نظر گرفتن این واقعیت که این عفونت به عنوان یکی از ریسک فاکتورهای مهم در افراد همودیالیزی است غربالگری‌های منظم و مستمر جهت تعیین آنتی‌بادی علیه توکسوپلاسما گوندی در این بیماران ضروری به نظر می‌رسد.

Title: آپی ژنین باعث بهبود عملکرد قلبی و کاهش سمیت قلبی ناشی از دوکسوروبیسین در موش صحرایی نر می شود

http://yafte.lums.ac.ir/article-1-2519-fa.html

مقدمه: دوکسوروبیسین در درمان بدخیمی­هایی شامل لنفوما، لوکمیا و سرطان پستان استفاده می­شود. سمیت قلبی، عارضه جانبی اصلی این دارو است. آپی­ژنین یک فلاونوئید دارای خواص آنتی­اکسیدانی، ضد التهابی و ضد توموری است. هدف این مطالعه بررسی اثرات آپی­ژنین بر سمیت قلبی ناشی از دوکسوروبیسین است. مواد و روش ­ها: 60 سر موش صحرایی نر ویستار در 6 گروه تقسیم شدند. سمیت قلبی با تزریق 6 نوبت دوکسوروبیسین         (mg/kg, ip2) در 12 روز ایجاد گردید. گروه­های تحت درمان همزمان با القای مدل سمیت قلبی، آپی­ژنین را با دوزهای mg/kg 25، 50 و 75 به صورت خوراکی به مدت 12 روز دریافت کردند. یافته­ ها: EF و FS در گروه آپی‌ژنین (mg/kg 25) نسبت به گروه سمیت قلبی افزایش معنی­دار (05/0>P) و LVEDs، LDH و CK-MB در گروه آپی‌ژنین (mg/kg 25) نسبت به گروه سمیت قلبی کاهش معنی­دار نشان داد (05/0>P). بحث و نتیجه­ گیری: آپی­ژنین مانع افزایش LDH و CK-MB، جلوگیری از کاهش میزان EF و FS و بهبود تغییرات بافتی قلب شده و باعث کاهش آسیب ناشی از سمیت قلبی توسط دوکسوروبیسین می­ گردد.

Title: اثرات تعاملی تمرین استقامتی و ژل آلوئه ورا بر آلانین آمینوترانسفراز و آسپارتات آمینوترانسفراز موش‌های صحرایی دیابتی

http://yafte.lums.ac.ir/article-1-2515-fa.html

مقدمه: فعالیت ورزشی و تغذیه دو رکن اساسی کنترل سطوح افزایش یافته آلانین آمینوترانسفراز (ALT) و آسپارتات آمینوترانسفراز (AST) در بیماری دیابت می­ باشند. هدف از تحقیق حاضر بررسی اثرات تعاملی تمرین استقامتی و ژل آلوئه­ ورا بر ALT و AST  موش­های صحرایی دیابتی بود. مواد و روش‌ها: 45 سر موش صحرایی دیابتی انتخاب و در پنج گروه 9 سری (1) کنترل هفته اول، (2) کنترل هفته ششم، (3) تمرین استقامتی، (4) مصرف آلوئه­ ورا و (5) تمرین استقامتی همراه با مصرف آلوئه ­ورا تقسیم‌ بندی شدند. همچنین جهت بررسی اثرات القای دیابت بر ALT و AST، 18 سر موش صحرایی در دو گروه (6) کنترل هفته اول و (7) کنترل هفته ششم قرار گرفتند. گروه­ های 3 و 5 به مدت شش هفته، 3 جلسه در هفته و هر جلسه 20 دقیقه روی نوارگردان دویدند. گروه­ های 4 و 5 روزانه mg/kg 100 ژل آلوئه ­ورا دریافت نمودند. تجزیه و تحلیل یافته ­های تحقیق با آزمون آنالیز واریانس یک‌ طرفه همراه با آزمون تعقیبی توکی صورت گرفت. سطح معنی‌داری کمتر از 05/0 در نظر گرفته شد (05/0P <). یافته­ها: القای دیابت اثر معنی‌داری بر افزایش ALT دارد (04/0=P)؛ تمرین استقامتی همراه با مصرف ژل آلوئه­ورا اثر معنی‌داری بر کاهش ALT و AST دارد (001/0=P)؛ تمرین استقامتی همراه با ژل آلوئه ­ورا نسبت به تمرین استقامتی و مصرف ژل آلوئه ­ورا اثر بیشتری بر کاهش ALT دارد (001/0=P) همچنین تمرین استقامتی همراه با ژل آلوئه ­ورا نسبت به تمرین استقامتی اثر بیشتری بر کاهش AST دارد (04/0=P). بحث و نتیجه‌ گیری: شش هفته تمرین استقامتی و ژل آلوئه­ ورا دارای اثرات تعاملی در بهبود ALT و AST موش­های دیابتی می‌باشد.

Title: تأثیر هشت هفته تمرین پیلاتس بر سطوح سرمی اورکسین و مقاومت به انسولین در کودکان دارای اضافه‌وزن

http://yafte.lums.ac.ir/article-1-2527-fa.html

مقدمه: نورون‌های اورکسین در پاسخ به استرس موجب برانگیختن و رفتار انگیزه می‌شوند. هدف از تحقیق حاضر بررسی اثر هشت هفته تمرین پیلاتس بر سطوح سرمی اورکسین و مقاومت به انسولین در کودکان دارای اضافه‌ وزن بود. مواد و روش­ ها: 20 نفر از کودکان دارای اضافه‌ وزن (13-10 سال) از بین افراد داوطلب شده به‌ صورت هدفمند انتخاب و به‌ صورت تصادفی در دو گروه تمرین پیلاتس (10 نفر) و کنترل (10 نفر) تقسیم شدند. ویژگی‌های فیزیولوژیک در هر دو گروه اندازه‌گیری شد. نمونه‌ های خون قبل و بعد از هشت هفته برنامه تمرینی پیلاتس جهت اندازه‌ گیری متغیرهای تحقیق اخذ شد. از آزمون t وابسته و مستقل جهت بررسی تغییرات درون‌ گروهی و بین گروهی متغیرهای تحقیق استفاده شد. یافته­ ها: بر اساس نتایج آزمون t همبسته مشاهده شد که تمرین پیلاتس باعث افزایش معنادار اورکسین شد (001/0P=). همچنین مشخص گردید که هشت هفته تمرین پیلاتس باعث کاهش معنادار مقاومت به انسولین، انسولین و گلوکز سرم می‌شود (001/0P=)؛ اما در گروه کنترل مقاومت به انسولین (001/0P=)، انسولین (001/0P=) و گلوکز (027/0P=) افزایش معناداری داشت. علاوه بر این هشت هفته تمرین پیلاتس تأثیر معناداری بر وزن بدن (001/0P=)، درصد چربی (001/0P=) و شاخص توده بدن (001/0P=) داشته است. همچنین مشخص گردید که در گروه کنترل وزن بدن، درصد چربی و شاخص توده بدنی افزایش معناداری داشته است. بحث و نتیجه­ گیری: یافته‌ها نشان می‌دهد که هشت هفته تمرین پیلاتس در افزایش اورکسین سرم، کاهش مقاومت به انسولین، انسولین و گلوکز سرم و همچنین بهبود ترکیب بدنی کودکان دارای اضافه‌ وزن مؤثر است.

Title: بررسی اثر دوز پایین نالوکسان روی شدت درد و عوارض جانبی اوپیوئیدها در بیماران تحت کوله سیستکتومی لاپاروسکوپیک

http://yafte.lums.ac.ir/article-1-2630-fa.html

مقدمه: مطالعات قبلی اثر نالوکسان روی شدت درد و عوارض جانبی اوپیوئیدها بعد از جراحی را بررسی کرده اند با این حال نتایج مطالعات متناقض است. مطالعه حاضر به منظور بررسی اثر تجویز دوز پایین نالوکسان روی شدت درد و میزان تهوع، استفراغ، خارش و احتباس ادراری در بیماران تحت کوله سیستکتومی لاپاروسکوپیک طراحی گردید.          مواد و روش ها: در مطالعه حاضر 60 بیمار نیازمند به عمل جراحی کوله سیستکتومی لاپاروسکوپیک شرکت نمودند و بعد از جراحی به طور تصادفی دو گروه تقسیم شدند: گروه نالوکسان (25/0 میکروگرم بر کیلوگرم در هر ساعت نالوکسان به علاوه 20 میکروگرم بر کیلوگرم در هر ساعت مورفین) و گروه دارونما (سرعت 20 میکروگرم بر کیلوگرم در هر ساعت مورفین). بیماران 2، 4، 8 و 16 ساعت بعد از ورود به ریکاوری ارزیابی شدند و شدت درد و بروز عوارض جانبی شامل تهوع، استفراغ، خارش  و احتباس ادراری توسط یک پرستار ثبت شد. یافته ها: شیوع تهوع، استفراغ، خارش و احتباس ادراری 2، 4، 8 و 16 ساعت بعد از جراحی به طور معنی داری در گروه نالوکسان کمتر از گروه دارونما بود. شدت درد بعد از جراحی بین دو گروه تفاوت معنی داری نداشت. بحث و نتیجه گیری: یافته های مطالعه حاضر نشان دادند که تجویز همزمان نالوکسان و مورفین در بیماران تحت کوله سیستکتومی لاپاروسکوپیک به طور معنی داری عوارض ناشی از اوپیوئیدها شامل تهوع، استفراغ، خارش و احتباس ادراری را در مقایسه با مورفین به تنهایی کاهش داد ولی تاثیری بر شدت درد بعد از جراحی نداشت.
